# Synergistic Regulating Mechanism of CLDH on the Mechanical Properties and Chloride Diffusion Behavior of Geopolymers

**DOI:** 10.3390/ma19091752

**Published:** 2026-04-24

**Authors:** Xu Gong, Xinchi Xu, Yuning Wu, Zhiji Gao, Gonghui Gu

**Affiliations:** 1 School of Design and Architecture, Zhejiang University of Technology, Hangzhou 310014, China; m13758233692@163.com; 2 College of Civil Engineering, Zhejiang University of Technology, Hangzhou 310014, China; 221123060161@zjut.edu.cn (X.X.); 302023524012@zjut.edu.cn (Y.W.); 3 Guangdong Provincial Key Laboratory of Durability for Marine Civil Engineering, Shenzhen Durability Center for Civil Engineering, Shenzhen University, Shenzhen 518060, China; 4 Zhejiang Key Laboratory of Green Construction and Intelligent Operation & Maintenance for Coastal Infrastructure, Zhejiang University of Technology, Hangzhou 310014, China

**Keywords:** CLDH, geopolymer, microstructural evolution, mechanical properties, chloride diffusion

## Abstract

Geopolymers have attracted increasing attention as sustainable binders, but their long-term durability in chloride-rich environments remains a critical concern. To elucidate the mechanistic role of calcined layered double hydroxides (CLDHs) in regulating the mechanical properties and chloride diffusion behavior of geopolymers, geopolymer pastes containing different CLDH contents were prepared. The compressive strength and chloride diffusion coefficient were determined, and the underlying mechanism was analyzed from the perspectives of geopolymerization degree, gel structure development, and pore structure evolution. The results indicate that the incorporation of CLDHs can promote geopolymerization, which may be associated with a nano-seeding effect, increasing the amount and degree of polymerization of the gel phases, refining the pore structure, and reducing pore connectivity. As a result, the compressive strength increases from 38.1 MPa to 49.2 MPa, while the chloride diffusion coefficient decreases by approximately 31.7% when the CLDH content reaches 6 wt.%. However, when the CLDH content exceeds this level, particle agglomeration limits effective gel growth, leading to microstructural deterioration and a weakened regulating effect.

## 1. Introduction

Geopolymers are a class of inorganic binders synthesized from aluminosilicate-rich solid wastes, such as mine tailings and rice husk ash, which are activated under alkaline or acidic environments [1,2]. They are characterized by a three-dimensional gel network structure and exhibit ceramic-like characteristics [3,4]. Previous studies have reported that, compared with conventional ordinary Portland cement (OPC) concrete, geopolymer concrete can reduce greenhouse gas emissions and energy consumption during production by approximately 73% and 43%, respectively [5]. In addition to their environmental advantages, geopolymers also exhibit superior resistance to aggressive marine environments compared with OPC-based materials [6]. Specifically, the relatively dense microstructure of geopolymers can effectively inhibit chloride penetration, thereby reducing the risk of reinforcement corrosion [7,8]. Moreover, the pore solution in geopolymers typically possesses higher alkalinity, which provides enhanced passivation protection for embedded steel reinforcement and delays the initiation of corrosion [9,10]. However, during long-term service in chloride-containing environments, chloride ions can still migrate through the internal pore structure of geopolymers and accumulate within the matrix [11], eventually triggering reinforcement corrosion and associated durability problems.

Previous studies have shown that geopolymer matrices possess a certain capacity for chloride binding [12,13]. Specifically, the complex three-dimensional molecular network of N-A-S-H (sodium aluminosilicate hydrate) gel contributes to enhancing the chloride-binding potential of geopolymer systems [14]. Ismail et al. [15] reported that N-A-S-H gel exhibits a larger specific surface area than C-A-S-H (calcium aluminosilicate hydrate) gel in fly ash-based geopolymer concrete, indicating a greater capability for chloride adsorption and encapsulation. However, the chloride-binding mechanism of N-A-S-H gel is dominated primarily by physical adsorption, which partially limits the chloride-binding capacity of geopolymers [16,17,18]. To address this limitation, previous studies have suggested that incorporating layered double hydroxides (LDH) into geopolymer systems can enhance chloride-binding capacity and contribute to suppressing chloride diffusion [19]. LDHs are a class of inorganic functional materials with a layered structure. Owing to their relatively weak interlayer electrostatic interactions and high specific surface area, LDHs can immobilize chloride ions through interlayer anion exchange and surface charge adsorption [20,21]. Nevertheless, the direct incorporation of LDH into geopolymer systems still presents certain limitations. On the one hand, LDH often exhibits poor dispersion within the matrix, which weakens its chloride-binding effectiveness and may even compromise the structural integrity of the material [22]. On the other hand, uncalcined LDH possesses limited reactivity and tends to act mainly as a physical filler during geopolymerization, making it difficult to continuously regulate gel structure evolution. In contrast, calcined layered double hydroxides (CLDH) exhibit a structural memory effect and can undergo rehydration reconstruction in alkaline environments to restore hydrotalcite-like structures, thereby providing higher reactivity and more stable anion-exchange capacity [23,24,25]. Therefore, compared with the direct incorporation of LDH, the use of CLDH is more favorable for simultaneously suppressing chloride transport and improving the mechanical performance of geopolymers. However, the synergistic regulation mechanism of CLDH on both the mechanical properties and chloride transport behavior of geopolymers, particularly from the perspective of coupled geopolymerization, gel evolution, and pore structure development, remains insufficiently understood. A systematic investigation is therefore still required.

In this study, CLDH-modified geopolymer paste was used as the research object. Geopolymer systems with different CLDH contents were designed, and a combination of reaction product analysis, pore structure characterization, mechanical performance testing, and chloride transport evaluation was employed to investigate the synergistic role of CLDH in promoting geopolymerization, enhancing mechanical properties, and suppressing chloride transport. Particular attention was given to clarifying the critical threshold at which the effect of CLDH transitions from reaction promotion to agglomeration-induced deterioration, as well as the underlying mechanisms associated with this transformation. Through this approach, a threshold-dependent regulation mechanism of CLDH is systematically elucidated, and the intrinsic linkage between geopolymerization, microstructural evolution, and macroscopic performance is established. The findings of this study are expected to provide a theoretical basis for the further application of CLDH in geopolymer materials used in marine engineering environments.

## 2. Methodology

### 2.1. Raw Materials and Mix Design

Metakaolin (MK), ground granulated blast furnace slag (GBFS), and magnesium–aluminum layered double hydroxide (LDH, Mg-Al-CO_3_ type) were used as the raw materials in this study. The LDH was purchased from Shanghai Macklin Biochemical Co., Ltd., Shanghai, China. The densities of MK, GBFS, and LDH were 2.70 g/cm^3^, 3.10 g/cm^3^, and 2.00 g/cm^3^, respectively. The chemical compositions of MK, GBFS, and LDH were determined by X-ray fluorescence (XRF) analysis, and the results are listed in Table 1. The XRF samples were oven-dried, ground to a fine powder, and prepared as pressed powder pellets. The XRF measurements were carried out at a tube voltage of 40 kV and a current of 100 μA. The alkali activator with a modulus (SiO_2_/Na_2_O molar ratio) of 0.9 was prepared by mixing sodium hydroxide (NaOH) with a sodium silicate solution. The NaOH was supplied as white granular particles with a purity greater than 96%. The sodium silicate solution possessed a silicate modulus (SiO_2_/Na_2_O molar ratio) of 3.30 and a Baumé degree of 38.5 °Bé, with Na_2_O and SiO_2_ mass fractions of 8.54 wt.% and 27.3 wt.%, respectively. The LDH was calcined in a muffle furnace by heating to 500 °C at a rate of 5 °C/min and holding for 3 h, followed by natural cooling to room temperature to obtain calcined layered double hydroxide (CLDH). The water-to-solid ratio of the base geopolymer system was fixed at 0.30, and the alkali equivalent was 6%. The total solid content of the base geopolymer system (MK, GBFS, and activator solids) was kept constant at 500 g, while CLDH was introduced as an external additive. The CLDH dosage was set approximately at 0%, 2%, 4%, 6%, 8%, and 10% of the total solid mass. The detailed mix proportions are presented in Table 2.

### 2.2. Specimen Preparation

All specimens were prepared according to the mix proportions listed in Table 2. MK, GBFS, and CLDH were first weighed according to the designed proportions and dry-mixed in a mechanical mixer at 60 rpm for 3 min. Subsequently, the composite alkali activator and mixing water were added, and wet mixing was conducted at 60 rpm for 2 min, followed by 120 rpm for an additional 2 min to obtain a homogeneous geopolymer paste. The fresh paste was then cast into 40 mm × 40 mm × 40 mm cubic molds and Φ50 mm × 100 mm cylindrical molds, followed by compaction on a vibrating table. The specimens were cured at 20 °C and 95% relative humidity for 24 h, then demolded and further cured under the same conditions for 28 days.

### 2.3. Test Methods

The compressive strength was measured in accordance with the Chinese standard GB/T 17671-2021 [26], which specifies the test method for cement mortar strength. The specimen size was 40 mm × 40 mm × 40 mm, and a force-controlled loading rate of 1 kN/s was applied. The average value of three parallel specimens was taken as the final result. The chloride diffusion performance was evaluated using a natural immersion method. Cylindrical specimens cured for 28 days were sealed with epoxy resin on all surfaces except the exposed erosion surface and then immersed in a 3.5% NaCl solution for 90 days. Powder samples were ground layer by layer along the penetration direction, and the chloride content was determined. The chloride diffusion coefficient was subsequently calculated based on Fick’s second law. Fourier transform infrared spectroscopy (FTIR) was conducted using a Nicolet iS50 spectrometer (Thermo Fisher Scientific, Waltham, MA, USA) over a scanning range of 400–4000 cm^−1^ with a resolution of 4 cm^−1^. Thermogravimetric (TG) analysis was performed using a TG 209 F3 instrument (NETZSCH, Selb, Germany) by heating the samples from room temperature to 800 °C at a rate of 10 °C/min under a nitrogen atmosphere. The internal moisture state of the specimens and their distribution in different pore and gel environments were analyzed using low-field nuclear magnetic resonance (^1^H NMR, MesoMR23-060H-I, Niumag, Suzhou, China). The T_2_ relaxation spectra were obtained using the CPMG pulse sequence. The pore structure parameters were determined by mercury intrusion porosimetry (MIP, AutoPore IV 9500, Micromeritics, Norcross, GA, USA), while the microstructural morphology was observed using scanning electron microscopy (SEM, Quanta 250 FEG, FEI, Hillsboro, OR, USA). For SEM observation, thin slices were cut from the central part of the specimens after compressive testing, with a lateral size of approximately 10 mm. The samples were then dried and sputter-coated with gold prior to observation.

Compressive strength and chloride diffusion tests were conducted on three parallel specimens, and the reported results represent the average values. Microstructural characterization techniques (including FTIR, TG, NMR, MIP, and SEM) were performed on representative samples.

## 3. Results and Discussions

### 3.1. Effect of CLDH Dosage on Mechanical Properties and Resistance to Chloride Penetration

**Figure 1:**
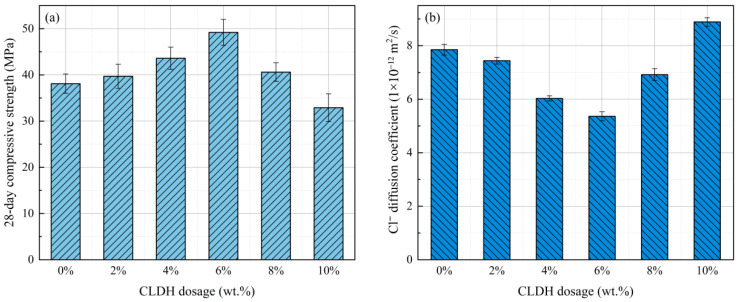
Twenty-eight-day compressive strength and Cl^−^ diffusion coefficient of geopolymer with different CLDH contents. (**a**) 28-day compressive strength; (**b**) Cl^−^ diffusion coefficient.

Figure 1a illustrates the evolution of the 28-day compressive strength of geopolymer specimens with different CLDH dosages. As the CLDH content increases from 0 wt.% to 6 wt.%, the compressive strength rises from 38.1 MPa to 49.2 MPa, indicating that an appropriate amount of CLDH can significantly enhance the mechanical performance of geopolymers. However, as the CLDH dosage continues to increase, the compressive strength exhibits a noticeable decreasing trend. Figure 1b presents the chloride diffusion coefficients of geopolymer specimens with different CLDH contents. The reference specimen shows a diffusion coefficient of 7.85 × 10^−12^ m^2^/s. With increasing CLDH dosage, the diffusion coefficient gradually decreases and reaches 5.36 × 10^−12^ m^2^/s at a CLDH content of 6 wt.%, representing a reduction of approximately 31.7% compared with the reference sample. This result indicates that the incorporation of CLDH can effectively suppress chloride transport in the geopolymer matrix. However, when the CLDH content exceeds 6 wt.%, the chloride diffusion coefficient increases again, reaching 6.92 × 10^−12^ m^2^/s and 8.89 × 10^−12^ m^2^/s at 8 wt.% and 10 wt.%, respectively. This increase suggests a deterioration in the resistance of the material to chloride penetration at excessive CLDH dosages.

Overall, by comparing the results of compressive strength and chloride diffusion, it can be observed that both properties exhibit similar variation trends with increasing CLDH dosage, reaching their optimal performance at approximately 6 wt.%. This observation suggests that the regulatory effect of CLDH on geopolymer performance exhibits a clear threshold characteristic. An appropriate amount of CLDH can simultaneously improve the mechanical properties and durability of the material, whereas excessive incorporation may adversely affect the structural integrity and transport properties of the geopolymer matrix. The evolution of these macroscopic properties is closely related to the influence of CLDH on the geopolymerization process and microstructural development. The underlying mechanisms will be further analyzed in the following sections.

### 3.2. Effect of CLDH on the Geopolymerization Degree and Gel Structure

**Figure 2:**
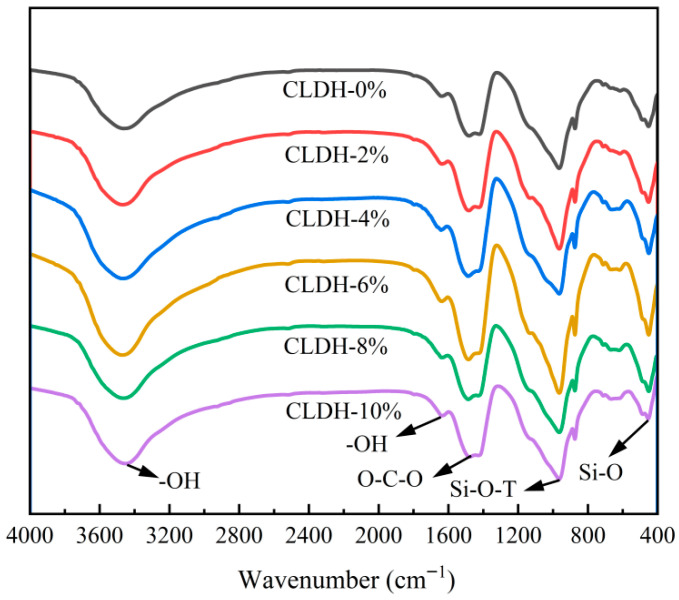
FTIR spectra of geopolymer with different CLDH contents.

Figure 2 presents the FTIR spectra of geopolymer specimens with different CLDH dosages. The characteristic peak at approximately 1485 cm^−1^ is associated with the vibration of O-C-O groups, indicating that carbonation occurred in the system during the curing process. The peaks at 3456 cm^−1^ and 1648 cm^−1^ correspond to the asymmetric stretching and bending vibrations of O-H bonds in bound water, respectively. The peak located near 450 cm^−1^ is related to the Si-O vibration in C-(A)-S-H gel. The broad absorption band in the range of 700–1300 cm^−1^ is attributed to the asymmetric stretching vibration of Si-O-T (T = Si or Al) bonds in N-A-S-H gel, which is an important characteristic region for evaluating gel formation and structural evolution [27]. Table 3 further summarizes the integrated peak areas within the 700–1300 cm^−1^ band. As the CLDH dosage increases from 0 wt.% to 6 wt.%, the peak area of this band gradually increases and reaches its maximum at 6 wt.%, indicating that the amount of gel products formed is highest at this dosage. However, when the CLDH content is further increased, the peak area decreases, suggesting that excessive CLDH is unfavorable for the continuous formation of gel products. This trend is consistent with the macroscopic results reported in Section 3.1, where both the compressive strength and the resistance to chloride diffusion reach optimal performance at a CLDH dosage of approximately 6 wt.%.

Previous studies have reported that CLDHs can undergo surface hydroxylation in highly alkaline environments and subsequently experience rehydration reconstruction, leading to the formation of a hydrotalcite-like (Ht) phase. The resulting reaction products may provide heterogeneous nucleation sites, thereby facilitating the precipitation and growth of C(N)-A-S-H gel and accelerating the overall reaction process of the geopolymer system [28,29]. Therefore, within an appropriate dosage range, the promoting effect of CLDH on gel formation can be effectively manifested. However, when the CLDH content is further increased, the effective reconstruction degree becomes limited despite the higher dosage. This phenomenon can be attributed to intensified particle interactions and a deteriorated dispersion state at high CLDH content, which reduces the effective reaction and reconstruction efficiency of CLDHs in the system, ultimately leading to a decrease in the amount of gel products formed.

**Figure 3:**
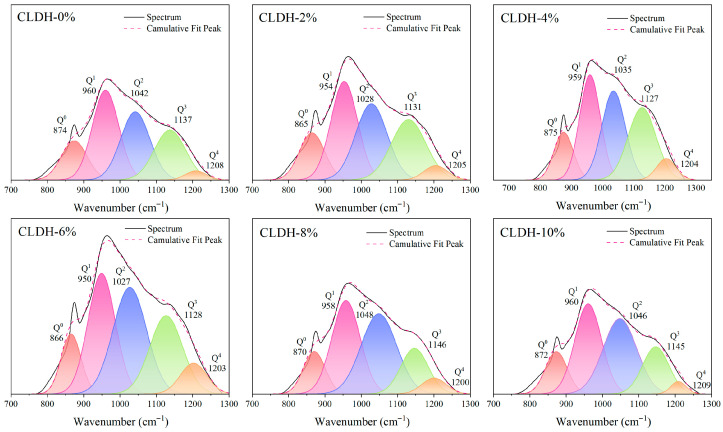
Deconvolution of the 700–1300 cm^−1^ band of geopolymer with different CLDH contents. The colored areas correspond to the deconvoluted peaks associated with Q^0^, Q^1^, Q^2^, Q^3^, and Q^4^ structural units, as indicated in the legend.

**Figure 4:**
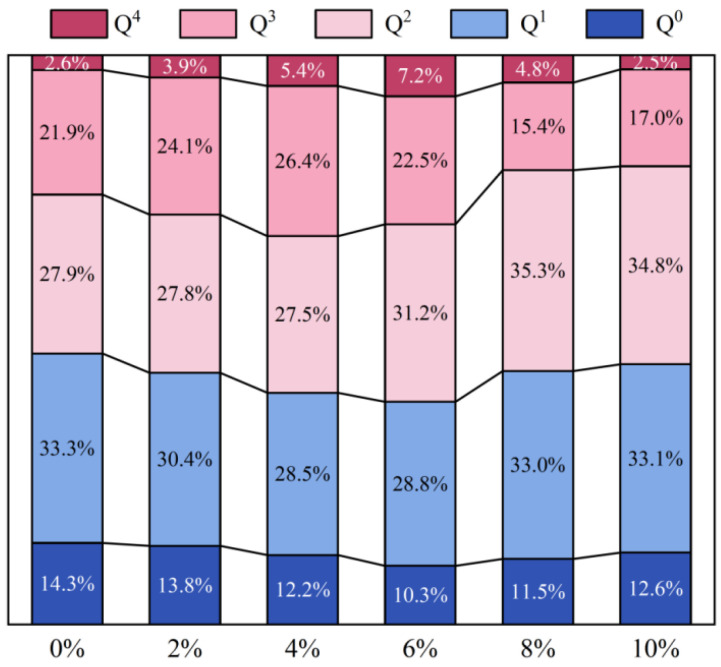
Distribution of Q^n^ species of geopolymer with different CLDH contents.

To further analyze the polymerization characteristics of the gel structure, Gaussian peak deconvolution was performed on the 700–1300 cm^−1^ band, and the fitting results are presented in Figure 3. This band can be decomposed into five components (Q^0^–Q^4^), which correspond to different connectivity states of SiO_4_ tetrahedra, ranging from monomeric units to highly cross-linked network structures [30]. The relative distribution of each Q^n^ component is shown in Figure 4. As the CLDH dosage increases from 0 wt.% to 6 wt.%, the proportions of the low-polymerization components (Q^0^ and Q^1^) decrease significantly, whereas the combined fraction of the highly polymerized components (Q^2^–Q^4^) increases from 52.4% to 60.9%, indicating that the degree of polymerization and cross-linking of the gel network is enhanced. However, when the CLDH content exceeds 6 wt.%, the proportion of highly polymerized components decreases, while the fractions of Q^0^ and Q^1^ increase, suggesting that excessive CLDH is unfavorable for the formation of highly cross-linked gel structures. Combined with the FTIR-integrated peak-area results, it can be concluded that the 6 wt.% CLDH dosage not only corresponds to a higher gel yield but also promotes the formation of a more highly polymerized gel network, which provides a microstructural basis for the simultaneous enhancement of mechanical strength and resistance to chloride penetration.

**Figure 5:**
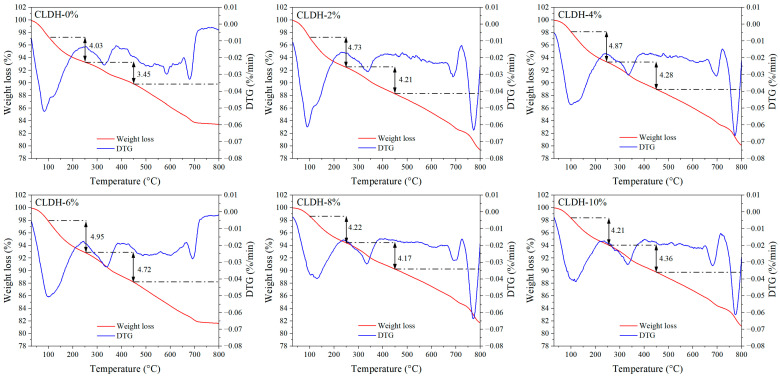
TG-DTG curves of geopolymer with different CLDH contents.

Figure 5 further presents the TG-DTG curves of geopolymer specimens with different CLDH dosages. The DTG curves reveal three main mass-loss stages. The initial mass loss below 250 °C corresponds to the removal of physically adsorbed and chemically bound water associated with N-A-S-H and C-(A)-S-H gels [31]. The mass loss within the temperature range of 250–450 °C is mainly attributed to the decomposition of hydrotalcite-like phases, accompanied by the release of interlayer water, hydroxyl groups, and partial anions during the decomposition process [32]. The mass-loss peak in the range of 500–800 °C reflects the decomposition of carbonate phases. Poorly crystalline carbonates typically decompose at 500–600 °C, whereas well-crystallized calcite exhibits a characteristic decomposition peak at approximately 700 °C [33]. It should be noted that the dehydroxylation reactions of C-(A)-S-H and N-A-S-H gels may persist up to about 600 °C [34]. However, the mass-loss behavior above 250 °C is simultaneously influenced by both hydrotalcite-like phases and carbonate phases, making it difficult to directly evaluate gel content solely based on the mass loss within the 250–600 °C range. Previous studies have indicated that the loss of bound water in C-(A)-S-H and N-A-S-H gels is mainly concentrated within the first DTG peak [32,35]. Considering that physically adsorbed water is primarily removed below 100 °C, while gel-related dehydration mainly occurs within the 100–250 °C range, the mass loss within 100–250 °C was selected in this study as a quantitative indicator of gel formation. The results show that the mass loss within this temperature range first increases and then decreases with increasing CLDH dosage, reaching a maximum at 6 wt.%, which is consistent with the gel formation and structural evolution trends revealed by the FTIR integrated peak area and Q^n^ structural analysis. Further analysis of the mass loss in the 250–450 °C range shows that it also reaches a relatively high value at a CLDH dosage of 6 wt.%, indicating that the rehydration reconstruction of CLDHs is more pronounced at this dosage. When the CLDH content exceeds 6 wt.%, the mass loss within this temperature range no longer increases with increasing CLDH dosage but instead decreases, suggesting that the effective rehydration reconstruction of CLDHs is inhibited under excessive dosage conditions.

**Figure 6:**
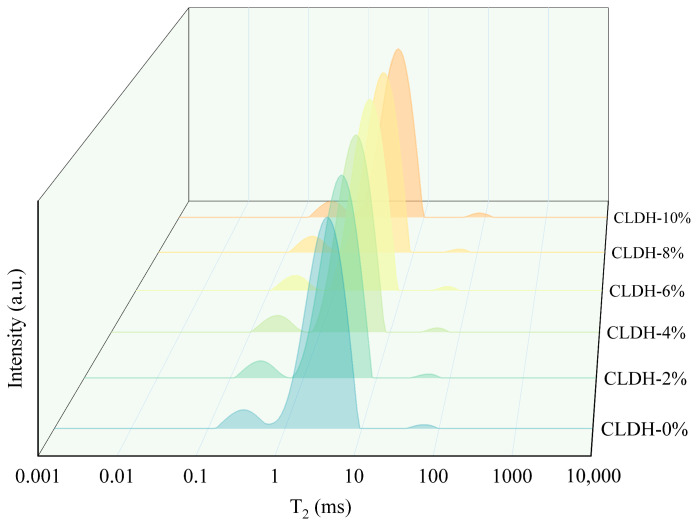
^1^H NMR T_2_ relaxation time distributions of geopolymer with different CLDH contents. Different colors represent samples with varying CLDH contents from 0% to 10%, as indicated on the right side of the figure.

Figure 6 shows the ^1^H NMR T_2_ relaxation time distributions of geopolymer specimens with different CLDH dosages at 28 days. According to previous studies, the T_2_ relaxation spectrum can generally be divided into four characteristic regions [36]: The range of 0.01–0.1 ms corresponds to interlayer water within gel structures, the range of 0.1–1 ms is associated with gel pore water, the range of 1–100 ms is related to capillary pore water, and the range of 100–10,000 ms corresponds to surface water. Due to the precision limitations of the testing instrument, the T_2_ relaxation signals in the range of 0.01–0.1 ms, which are associated with interlayer water in gels and CLDH crystalline structures, could not be effectively captured in this study. It can be observed that as the CLDH dosage increases from 0 wt.% to 6 wt.%, the characteristic peaks shift toward shorter relaxation times. When the CLDH content exceeds 6 wt.%, the peaks shift back toward longer relaxation times. A reduction in relaxation time generally indicates that the pore water is subjected to stronger confinement, stronger interactions with the solid surface, or a higher degree of water binding. Conversely, an increase in relaxation time suggests a weaker confinement environment for pore water. Therefore, the ^1^H NMR results reflect a positive influence of CLDHs on the microstructural evolution of the geopolymer system at approximately 6 wt.%, as evidenced by enhanced confinement and binding state of pore water within the pore structure.

Overall, as the CLDH dosage increases from 0 wt.% to 6 wt.%, both the amount of gel products and the degree of gel polymerization increase simultaneously. However, when the CLDH content exceeds 6 wt.%, both gel formation and structural evolution exhibit a declining trend. This threshold behavior at the microstructural level is consistent with the macroscopic results presented in Section 3.1, where mechanical performance and resistance to chloride diffusion reach their optimal values at a CLDH dosage of 6 wt.%. These results suggest that the regulatory effect of CLDH on the reaction degree and gel structure of the system is one of the key factors governing variation in macroscopic properties.

### 3.3. Effect of CLDHs on Pore Structure

**Figure 7:**
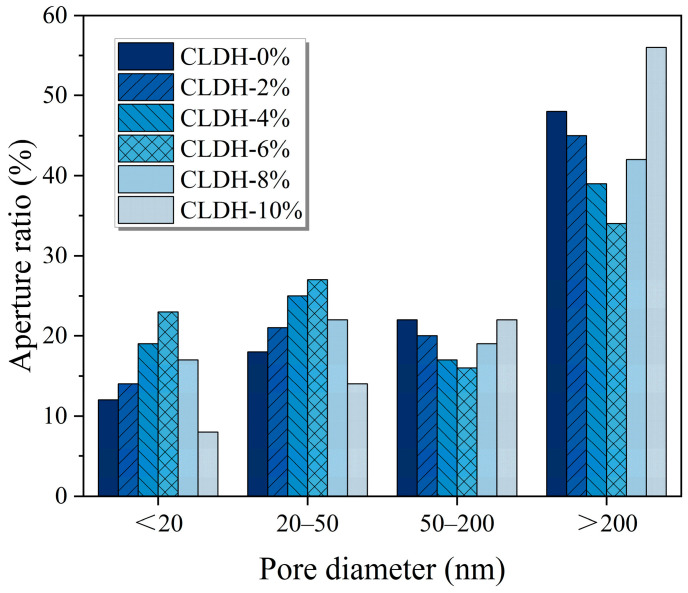
Pore size distribution of geopolymer with different CLDH contents.

Figure 7 shows the pore-size distribution characteristics of geopolymer specimens with different CLDH dosages obtained from MIP measurements. In the reference sample, the proportions of mesopores and macropores are relatively high, indicating a comparatively coarse pore structure. As the CLDH dosage increases to 6 wt.%, the pore size distribution shifts markedly toward the micropore and small-pore regions, while the volume fractions of mesopores and macropores decrease significantly, indicating a redistribution of pore sizes, characterized by a reduction in macropores and a shift toward finer pore regions. This change may be associated with the rehydration reconstruction of CLDH in the alkaline environment, which gradually leads to the formation of hydrotalcite-like (Ht) phases. On the one hand, the Ht phase can act as heterogeneous nucleation sites, promoting the precipitation and growth of C(N)-A-S-H gels. On the other hand, the nanoscale size of the Ht phase may contribute to filling gel interspaces and original pore spaces. Through the synergistic effects of reaction promotion and physical filling, the structural compactness of the system is significantly improved [37]. As the pore-size distribution shifts toward finer pores, both the pore connectivity and the characteristic size of transport pathways decrease markedly, thereby effectively suppressing the diffusion and migration of chloride ions within the matrix. However, when the CLDH dosage is further increased, excessive CLDH particles tend to agglomerate within the paste. Reaction products preferentially form on the outer surfaces of these agglomerates, generating dense encapsulation layers that restrict rehydration reconstruction and ion diffusion within the interior CLDH particles. As a result, the Ht phase cannot be uniformly distributed throughout the matrix, which weakens the precipitation and filling effects of gel products. Consequently, the pore structure in some regions becomes coarser again, and the proportion of connected pores increases. Correspondingly, the effective transport pathways for chloride ions within the material increase, leading to a reduction in the material’s physical barrier capacity against aggressive media.

**Figure 8:**
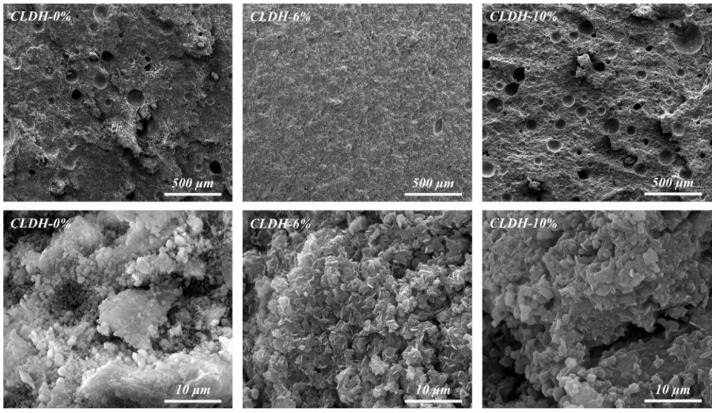
SEM micrographs of geopolymer with different CLDH contents.

The SEM images shown in Figure 8 further corroborate the pore structure evolution discussed above. When a CLDH dosage is 6 wt.%, the matrix exhibits the most compact and continuous microstructure, with significantly reduced pores and microcracks, forming a stable and continuous spatial skeleton. However, when the CLDH content is further increased, localized regions with looser structures, increased porosity, and the development of microcracks can be observed. These observations indicate that excessive CLDH is unfavorable for the sustained formation of a dense microstructure.

### 3.4. Discussion

The results of this study demonstrate a clear threshold-dependent regulation behavior of CLDH in geopolymer systems, where an optimal dosage exists for both mechanical performance and chloride transport resistance. Similar trends have been reported in previous studies on LDH-modified cementitious materials [38]. For instance, the incorporation of a small amount of CLDH was found to significantly enhance early-age strength, while excessive addition led to performance deterioration due to particle agglomeration [39]. However, the critical threshold values vary depending on raw materials, mix proportions, and reaction environments, indicating that the threshold behavior is system-dependent.

In comparison with conventional nanoparticle additives, such as nano-SiO_2_ or nano-CaCO_3_, CLDH exhibits both similarities and distinct features. Similar to nanoparticles, CLDH can contribute to pore structure refinement and matrix densification through physical filling effects and provide additional nucleation sites. More importantly, CLDH influences the geopolymerization process by promoting the formation and structural evolution of gel phases, leading to an increased degree of polymerization and a more compact gel network [40]. In addition, CLDH possesses a structural memory effect and ion-exchange capability, enabling it to participate in chemical interactions and contribute to chloride binding. Therefore, the regulation mechanism of CLDH involves coupled physical and chemical effects.

Furthermore, this study suggests a link between the geopolymerization degree, gel structure evolution, pore structure refinement, and macroscopic performance. The transition from performance enhancement to deterioration with increasing CLDH content can be attributed to the balance between gel formation and particle agglomeration. At low dosages, well-dispersed CLDH particles provide nucleation sites and promote geopolymerization, thereby increasing the amount and polymerization degree of gel phases and refining the pore structure, resulting in a denser matrix. In contrast, at higher dosages, particle agglomeration limits effective gel development and introduces structural heterogeneity, leading to increased pore connectivity and deterioration of both mechanical and transport properties.

## 4. Conclusions

This study systematically investigated the synergistic regulatory mechanism of calcined layered double hydroxides (CLDHs) on the mechanical properties and chloride diffusion behavior of geopolymers from the perspectives of geopolymerization, gel structure evolution, and pore structure development. The main conclusions are summarized as follows:

(1) CLDH exhibits a significant regulatory effect on both the mechanical properties and chloride transport resistance of geopolymers, with a clear dosage threshold characteristic. As the CLDH dosage increases from 0 wt.% to 6 wt.%, the 28-day compressive strength increases by 29.1%, while the chloride diffusion coefficient decreases by 31.7%, indicating a simultaneous enhancement of mechanical performance and resistance to chloride penetration. However, when the CLDH dosage is further increased, the improvement trend reverses, and the overall performance gradually deteriorates.
(2) The incorporation of CLDHs significantly regulates the geopolymerization process and gel structure development. Within the optimal dosage range (around 6 wt.%), CLDH can provide heterogeneous nucleation sites, promoting gel formation and increasing the degree of polymerization of gel networks, thereby contributing to the development of the gel structure toward a more highly polymerized and densified state. At higher dosages, particle agglomeration weakens the effective participation of CLDHs in the reaction, hindering the continued evolution of the gel structure toward a favorable densified configuration.
(3) At appropriate CLDH dosages, the pore size distribution shifts toward smaller pores, accompanied by a significant reduction in macropores and microcracks. As a result, the pore structure evolves toward lower connectivity and higher compactness, leading to enhanced structural continuity of the matrix. When the CLDH dosage exceeds the optimal level, particle agglomeration limits the effective precipitation and filling of gel products, causing the pore structure to transition from densification to heterogeneous coarsening, accompanied by an increase in structural defects.
(4) Within the appropriate dosage range, CLDH is considered to undergo rehydration reconstruction in alkaline environments to form hydrotalcite-like phases, which synergistically provide heterogeneous nucleation and nano-filling effects. These processes promote geopolymerization and drive the gel structure and pore structure toward a more compact and low-connectivity configuration, thereby achieving synergistic enhancement of mechanical properties and resistance to chloride attack. However, when the CLDH dosage exceeds the critical threshold, particle agglomeration weakens the rehydration reconstruction and nucleation effects of CLDH, leading to simultaneous deterioration of the gel structure, pore structure, and macroscopic performance.

## Figures and Tables

**Table 1:** The chemical composition of MK, GBFS, and LDH (wt.%).

Material	SiO_2_	Al_2_O_3_	Fe_2_O_3_	MgO	CaO	Na_2_O	K_2_O	MnO	TiO_2_	Loss
MK	50.37	46.48	-	-	0.35	0.86	0.21	0.10	0.93	0.70
GBFS	28.83	15.71	0.66	9.06	38.07	1.09	0.51	0.60	2.05	3.42
LDH	0.07	28.37	0.01	48.94	0.09	0.08	-	-	-	22.44

**Table 2:** The composition design of the geopolymer composite.

Mixture	Total Solid Content (g)	MK (g)	GBFS (g)	NaOH (g)	Water Glass (g)	Water (g)	CLDH (g)
CLDH-0%	500	224.8	224.8	25.3	86.1	89.1	0
CLDH-2%	500	224.8	224.8	25.3	86.1	89.1	10
CLDH-4%	500	224.8	224.8	25.3	86.1	89.1	20
CLDH-6%	500	224.8	224.8	25.3	86.1	89.1	30
CLDH-8%	500	224.8	224.8	25.3	86.1	89.1	40
CLDH-10%	500	224.8	224.8	25.3	86.1	89.1	50

**Table 3:** Peak area of the 700–1300 cm^−1^ band of geopolymer with different CLDH contents.

Mixture	CLDH-0%	CLDH-2%	CLDH-4%	CLDH-6%	CLDH-8%	CLDH-10%
Peak area (A.U.)	6454	7877	8100	10,168	7379	6230

## Data Availability

The original contributions presented in this study are included in the article. Further inquiries can be directed to the corresponding authors.
